# Necdin, a Negative Growth Regulator, Is a Novel STAT3 Target Gene Down-Regulated in Human Cancer

**DOI:** 10.1371/journal.pone.0024923

**Published:** 2011-10-27

**Authors:** Rachel Haviland, Steven Eschrich, Gregory Bloom, Yihong Ma, Susan Minton, Richard Jove, W. Douglas Cress

**Affiliations:** 1 Molecular Oncology, Moffitt Cancer Center and Research Institute, Tampa, Florida, United States of America; 2 Biomedical Informatics, Moffitt Cancer Center and Research Institute, Tampa, Florida, United States of America; 3 Breast Cancer Program, Moffitt Cancer Center and Research Institute, Tampa, Florida, United States of America; 4 Beckman Research Institute, City of Hope National Medical Center, Duarte, California, United States of America; Massachusetts General Hospital, United States of America

## Abstract

Cytokine and growth factor signaling pathways involving STAT3 are frequently constitutively activated in many human primary tumors, and are known for the transcriptional role they play in controlling cell growth and cell cycle progression. However, the extent of STAT3's reach on transcriptional control of the genome as a whole remains an important question. We predicted that this persistent STAT3 signaling affects a wide variety of cellular functions, many of which still remain to be characterized. We took a broad approach to identify novel STAT3 regulated genes by examining changes in the genome-wide gene expression profile by microarray, using cells expressing constitutively-activated STAT3. Using computational analysis, we were able to define the gene expression profiles of cells containing activated STAT3 and identify candidate target genes with a wide range of biological functions. Among these genes we identified Necdin, a negative growth regulator, as a novel STAT3 target gene, whose expression is down-regulated at the mRNA and protein levels when STAT3 is constitutively active. This repression is STAT3 dependent, since inhibition of STAT3 using siRNA restores Necdin expression. A STAT3 DNA-binding site was identified in the Necdin promoter and both EMSA and chromatin immunoprecipitation confirm binding of STAT3 to this region. Necdin expression has previously been shown to be down-regulated in a melanoma and a drug-resistant ovarian cancer cell line. Further analysis of Necdin expression demonstrated repression in a STAT3-dependent manner in human melanoma, prostate and breast cancer cell lines. These results suggest that STAT3 coordinates expression of genes involved in multiple metabolic and biosynthetic pathways, integrating signals that lead to global transcriptional changes and oncogenesis. STAT3 may exert its oncogenic effect by up-regulating transcription of genes involved in promoting growth and proliferation, but also by down-regulating expression of negative regulators of the same cellular processes, such as Necdin.

## Introduction

STAT3 is a latent cytoplasmic transcription factor, induced by a variety of upstream signals, including growth factors, cytokines and non-receptor tyrosine kinases [1]–[7]. Upon activation by tyrosine phosphorylation, STAT3 forms dimers, which translocate to the nucleus and regulate transcription of target genes. Under normal physiological conditions, STAT3 activity is tightly controlled; however, intracellular signaling pathways involving STAT3 are frequently constitutively activated in many different human primary tumors [8]–[15]. We and others have shown that constitutive activation of STAT3 provides cancer cells with growth and survival advantages [16] and enhances tumor angiogenesis [17] and metastasis [18]. Recent studies have also indicated that STAT3 activation contributes to tumor immune evasion [19], [20]. These findings indicate that aberrant STAT3 signaling affects a wide variety of fundamental cellular functions through multiple mechanisms.

To date, up-regulated expression of numerous STAT3 target genes has been identified, including VEGF [17], Bcl-2, Bcl-xL [21], p21, Cyclin D1 [22] and survivin [23]. These STAT3 target genes have generally been identified on an individual basis, while few studies have attempted to identify large numbers of STAT3 regulated genes [24]–[28]. We took a broad approach to identify novel STAT3 regulated genes involved in oncogenesis by examining changes in the genome-wide gene expression profile by microarray, using cells expressing constitutively-active STAT3. Combining this approach with computational analysis of the microarray results, we were able to define the gene expression profile of cells expressing activated STAT3 and examine the role of STAT3 in both positive and negative regulation of gene expression.

Pathway and functional analysis demonstrate that STAT3 has an important role in regulating, both positively and negatively, a diverse array of cellular processes in addition to transcription. STAT3 coordinates expression of genes involved in multiple metabolic and biosynthetic pathways, integrating signals that lead to global transcriptional changes and oncogenesis. These include genes involved in cell adhesion, cytoskeletal remodeling, nucleotide, lipid and protein metabolism, as well as signal transduction.

Through computational analysis of our data, we identified Necdin, a negative growth regulator, as a novel potential STAT3 target gene. Necdin is a potent growth suppressor that is predominantly expressed in post-mitotic neurons [29]–[32]. Necdin expression has been shown to be down-regulated in both carcinoma cell lines and primary tumors [33], suggesting that repression of Necdin expression may have a role in oncogenesis. We verified that Necdin mRNA expression inversely correlates with STAT3 activity in cells expressing constitutively-active STAT3 and that STAT3 directly regulates the expression of Necdin at the promoter level. In addition, Necdin expression in human tumor cell lines is inversely correlated with activation of endogenous STAT3. Our findings provide further evidence for a role of Necdin as a physiological target of STAT3, demonstrating that computational analysis of microarray data can be used to identify potential STAT3 target genes for further investigation.

## Results

### Identification of Potential STAT3 Target Genes Using Oligonucleotide Microarrays

To identify potential novel STAT3-regulated genes, we examined global gene expression patterns in cell lines harboring persistently active STAT3. Gene expression profiles in such cells are likely to be representative of the genetic profile of a cancer cell with aberrant STAT3 expression, as compared to inducing STAT3 activity transiently using exogenous stimulation, such as IL-6 or transient transfection [25].

RNA was harvested from normal Balb/c-3T3 cells with low levels of endogenous STAT3 activity, to serve as a control. RNA was also extracted from Balb/c-3T3 cells stably transfected with either v-Src, known to induce persistent activation of STAT3 [34], [35], or the constitutively active mutant, STAT3-C [36]. Triplicate samples were collected, one each from three consecutive passages, and each RNA sample was hybridized to a single Affymetrix Mouse Genome 430 2.0 GeneChip. Significance Analysis of Microarrays (SAM) [37] was used to identify differentially expressed genes between parental Balb/c-3T3 cells and cells stably transfected with either v-Src or STAT3-C. We accepted all genes identified by SAM as differentially regulated by at least 1.5-fold [38].

### Overlap of the Two Microarray Data Sets

Microarray analysis and subsequent SAM generated two lists of differentially expressed genes: one list identified genes differentially expressed between control Balb/c-3T3 cells and cells transfected with v-Src, and the second list contained genes differentially expressed between control Balb/c-3T3 cells and cells transfected with STAT3-C. Genes common to both lists are most likely to be directly regulated by STAT3. These genes were identified by cross-referencing the data in the two lists using the Microsoft Excel VLOOKUP function.

While v-Src transformed cells have constitutively active STAT3, Src also stimulates other STAT3-independent pathways [39]–[42]. In contrast, target genes activated by STAT3-C are limited to direct binding of the activated protein to STAT3 consensus sites in DNA. Therefore, using cells stably transfected with either v-Src or STAT3-C allowed us to control for clonal variations, as well as divergence in signaling pathways depending on the mechanism of STAT3 activation. The use of multiple microarray replicates in our approach further increases confidence in the results. This allowed us to identify a set of common genes as targets of STAT3. The data were further validated by the identification of several previously characterized STAT3-regulated genes, including CCND1, p21 [22], VEGFA [17], and Mcl-1 [43]. The most significantly over-expressed (induced) and under-expressed (repressed) genes are listed in Table 1 and Table 2, respectively. A more extensive list, including the top 50 significantly over-expressed and under-expressed genes are included in the Tables S1 and S2.

**Table 1:** Most Significant Probesets Over-Expressed Common to v-Src and STAT3-C.

				v-Src Data		STAT3-C Data		
Accession	Affy Probeset	Gene Name	Gene Description	Score(d)	Fold Change	Score(d)	Fold Change	Av. Score
NM_024223	1417311_at	Crip2	cysteine rich protein 2	51.343	17.4918	72.639	26.6976	61.991
NM_021451	1418203_at	Pmaip1	phorbol-12-myristate-13-acetate-induced protein 1	39.681	31.3643	34.058	47.1463	36.869
NM_009701	1418818_at	Aqp5	aquaporin 5	21.732	22.2635	45.193	94.0883	33.463
AF352788	1451527_at	Pcolce2	procollagen C-endopeptidase enhancer 2	36.947	19.9492	27.024	31.4550	31.986
AV066880	1452592_at	Mgst2	microsomal glutathione S-transferase 2	29.291	15.1149	34.651	37.3030	31.971
BF235516	1420842_at	Ptprf	protein tyrosine phosphatase, receptor type, F	16.848	20.1766	44.849	20.1217	30.848
NM_013867	1415936_at	Bcar3	breast cancer anti-estrogen resistance 3	26.309	11.7696	33.804	22.3994	30.056
BI251808	1416613_at	Cyp1b1	cytochrome P450, family 1, subfamily b, polypeptide 1	21.870	47.4091	36.953	84.8654	29.412
NM_053132	1449527_at	Pcdhb7	protocadherin beta 7	25.742	18.8861	29.613	16.6966	27.677
AF022072	1425458_a_at	Grb10	growth factor receptor bound protein 10	29.734	22.6049	23.021	30.5514	26.378
BB041811	1455900_x_at	Tgm2	transglutaminase 2, C polypeptide	33.990	5.1505	16.141	19.8117	25.065
NM_011577	1420653_at	Tgfb1	transforming growth factor, beta 1	38.605	4.2769	11.154	2.2493	24.879

The top genes ranked by Average Score identified by SAM as being upregulated in common by v-Src and STAT3-C.

**Table 2:** Most Significant Probesets Under-Expressed Common to v-Src and STAT3-C.

				v-Src Data		STAT3-C Data		
Accession	Affy Probeset	Gene Name	Gene Description	Score(d)	Fold Change	Score(d)	Fold Change	Av. Score
AF081260	1418070_at	Cdyl	chromodomain protein, Y chromosome-like	−72.232	0.0548	−47.865	0.0832	−60.049
NM_010882	1415923_at	Ndn	necdin	−58.571	0.0081	−54.087	0.0102	−56.329
AW743020	1435382_at	Ndn	necdin	−30.525	0.0135	−73.974	0.0122	−52.249
NM_009866	1450757_at	Cdh11	cadherin 11	−32.836	0.0055	−52.719	0.0048	−42.778
BB074430	1437853_x_at	Ndn	necdin	−16.328	0.1440	−67.114	0.1065	−41.721
AV228782	1434261_at	Sipa1l2	signal-induced proliferation-associated 1 like 2	−43.072	0.0715	−38.883	0.1002	−40.978
BB259670	1437284_at	Fzd1	frizzled homolog 1 (Drosophila)	−16.598	0.0997	−61.622	0.0879	−39.110
AW743020	1435383_x_at	Ndn	necdin	−40.515	0.0120	−29.383	0.0141	−34.949
BB125261	1448293_at	Ebf1	early B-cell factor 1	−46.495	0.0688	−19.975	0.0796	−33.235
NM_007993	1460208_at	Fbn1	fibrillin 1	−38.299	0.0180	−26.476	0.0200	−32.388
AV124445	1455792_x_at	Ndn	necdin	−30.296	0.0151	−32.708	0.0189	−31.502
NM_011581	1422571_at	Thbs2	thrombospondin 2	−33.293	0.0148	−26.721	0.0124	−30.007

The top genes ranked by Average Score identified by SAM as being downregulated in common by v-Src and STAT3-C.

To date, the majority of studies have examined putative STAT3 target genes which are up-regulated or over expressed when STAT3 is active. STAT3 has been shown to activate transcription of many genes involved in oncogenesis [8], cell survival [16], tumor progression [23] and metastasis [18]. STAT3 has also previously been shown to repress the transcription of a handful of genes, including p53 [44] and nitric oxide synthase [45] However, our results demonstrate that STAT3 is capable of repressing expression of a much larger number of genes. This novel discovery has the potential to profoundly impact the biology of cells harboring constitutively active STAT3.

One such gene which appears to be repressed by STAT3 is the negative growth regulator Necdin. Five Affymetrix probesets corresponding to NDN are ranked in the list of the top 12 most significantly repressed probesets (Table 2), suggesting that Necdin is significantly repressed when STAT3 is constitutively active. This demonstrates that computational analysis of microarray data can be used to identify potential STAT3 target genes for further investigation.

### Pathway Analysis Reveals Known and Novel Functions of STAT3

Microarrays assess simultaneous changes in transcript levels on an individual basis, resulting in a long list of genes which have significantly changed transcript levels when compared to control cells. However, these changes in gene expression do not occur as independent events within the cell, but are controlled in a coordinated manner and are often interconnected. Pathway Analysis is an unbiased method to determine whether differentially expressed genes, and the proteins they encode, are enriched in particular pathways, giving insight into the biological meaning of the changes observed.

We subjected the list of differentially expressed genes in common between v-Src and STAT3-C expressing cells to the MetaCore™ Analysis Suite (GeneGO) and compared them to known biological pathways in the MetaBase™ database. Using this analysis we were able to identify known STAT3 pathways, including the JAK/STAT pathway and Angiotensin/STAT pathway. This provides support for the use of such analyses to identify novel pathways that may also be regulated by STAT3.

Cell adhesion and cytoskeletal remodeling were among the most significantly enriched pathways identified from the differentially expressed genes (Table 3). The role of STAT3 in cytoskeletal remodeling has previously been reported [46]. Functional analysis of the genes we identified in cytoskeletal remodeling processes, indicates that STAT3 regulates genes involved in protein phosphorylation, signaling (MAPKK and Ras pathways), as well as response to hypoxia and cell migration.

**Table 3:** Enriched Pathways in Genes Differentially Expressed by v-Src and STAT3-C.

Cell process	Count	%	p-Value
Cell adhesion - Integrin-mediated cell adhesion and migration	19	42.22	5.56E-05
Cytoskeleton remodeling	32	33.33	6.09E-05
Development - Angiotensin signaling via STATs	13	52.00	6.25E-05
Cell adhesion, cytokine and chemokine mediated signaling pathway	31	33.33	7.93E-05
Transcription - Ligand-dependent Transcription of Retinoid Target Genes	15	46.88	8.03E-05
Proteolysis	12	50.00	1.97E-04
Cytoskeletal remodeling and cell adhesion - Integrin outside in signaling	18	39.13	2.79E-04
Development - WNT signaling, degradation of beta-catenin	10	50.00	6.83E-04
G-protein signaling - RhoA regulation pathway	14	41.18	7.26E-04
Immune response - IFN alpha/beta signaling pathway	11	45.83	9.32E-04

Over-represented pathways identified using the MetaCore Analysis Suite (GeneGO Inc.).

We also examined the genes regulated by STAT3 in cell adhesion and demonstrated that proteins involved in cell-matrix adhesion and cell-cell adhesion, particularly focal adhesion formation, were particularly enriched when STAT3 is constitutively active, as well as several genes in the integrin cell adhesion pathway. As such, we show that computational analysis of microarray data can identify both known and novel pathways regulated by STAT3.

### Functions of Induced Genes

To determine the functional classification of the differentially expressed genes identified by SAM, we performed Functional Annotation using the tool in the DAVID Bioinformatics Database (http://david.abcc.ncifcrf.gov/) [47], [48]. A wide range of target genes were altered by STAT3 activation, including genes involved in multiple pathways regulating biological and cellular processes, protein localization and transport, as well as organ and system development (Table 4). Genes within these categories (Table S3. ‘Affymetrix Probeset IDs’ column expands to list all probeset IDs) include many involved in cell growth and maintenance, such as lipid, nucleotide and protein synthesis, metabolism and/or localization (including VLDLR, APOL6, AK5, MTAP, UPP1, POP5, NUPL1, SEC61B, VDP) as well as signal transduction, all of which are required to promote cell growth and proliferation.

**Table 4:** Functional Enrichment (based on GO Biological Process) in Genes Differentially Expressed in Common by v-Src and STAT3-C using DAVID.

GO Category Level	GO Term	GO Category	Count	p Value
GOTERM_BP_3	GO:0050794	Regulation of cellular process	535	1.64E-14
GOTERM_BP_3	GO:0043283	Biopolymer metabolic process	627	5.89E-12
GOTERM_BP_3	GO:0048519	Negative regulation of biological process	170	3.05E-09
GOTERM_BP_3	GO:0008104	Protein localization	140	5.76E-09
GOTERM_BP_3	GO:0045184	Establishment of protein localization	131	9.26E-09
GOTERM_BP_3	GO:0048523	Negative regulation of cellular process	158	1.96E-08
GOTERM_BP_3	GO:0009653	Anatomical structure morphogenesis	187	2.40E-08
GOTERM_BP_3	GO:0015031	Protein transport	123	3.79E-08
GOTERM_BP_3	GO:0048513	Organ development	213	7.26E-08
GOTERM_BP_3	GO:0048731	System development	248	7.76E-07

The top 10 functionally enriched categories identified by DAVID ranked by p-Value.

STAT3 has a well characterized role in regulating gene transcription, however, we also show through Functional Analysis, that STAT3 controls the expression of genes involved in cellular processes required to transport the proteins and regulate their subcellular localization. This supports our hypothesis that STAT3 coordinates multiple pathways within the cell and reveals that STAT3 has wide-ranging effects, controlling multiple cellular pathways involved in fundamental biological processes. Our results suggest that STAT3 orchestrates transcription, translation, transport and localization leading to wide reaching effects on cell growth, proliferation and survival.

In contrast to previous studies of STAT3 target genes, we demonstrated that STAT3 regulates a diverse array of genes in both a positive and negative manner. Most genes regulated by STAT3 that have been identified to date demonstrate increased expression in cells where STAT3 is activated. However, our results also show that STAT3 signaling causes repression of many genes, including Necdin, which could profoundly impact the biology of cells harboring constitutively active STAT3.

### Constitutively Activated STAT3 Blocks Necdin mRNA Expression

**Figure 1:**
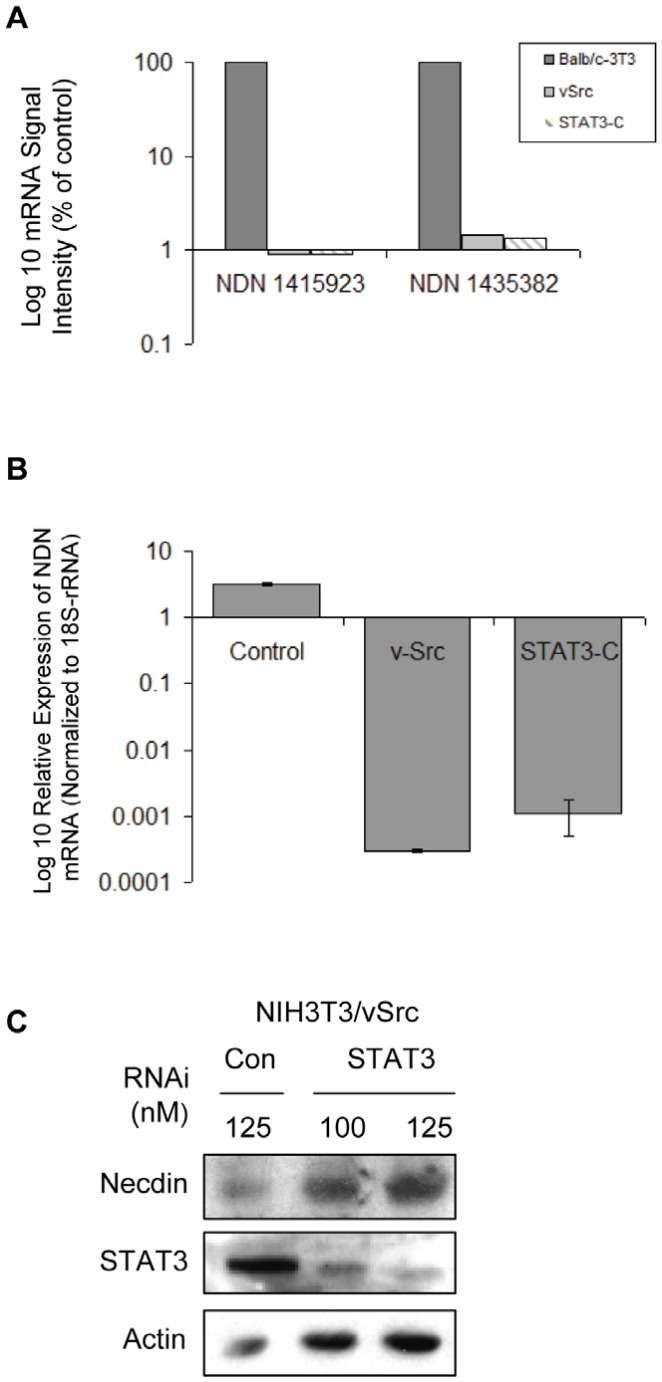
Analysis of Necdin expression in cell lines stably expressing v-Src or STAT3-C. **A.** Microarray Analysis of Necdin mRNA expression levels. RNA from Balb/c-3T3 cells stably expressing either pMvSrc or pRc-STAT3-C was isolated, processed and hybridized to Affymetrix Mouse Genome 430 2.0 GeneChips. In order to control for both biological and experimental variation, for each cell line, cells were harvested from three consecutive passages. At each passage, five 10 cm plates of exponentially growing cells were pooled for RNA extraction and the RNA hybridized to a single Affymetrix Mouse Genome 430 2.0 GeneChip. The arrays were scanned and fluorescence was measured with an Affymetrix GeneArray 3000 GeneChip scanner and the image processed using the GeneChip Operating Software (GCOS) version 1.1 to produce CEL files containing probe-level data for each GeneChip. All microarray experiments were done in triplicate independent experiments, and the results are presented for each probe set as average fold change in RNA expression. Data for two different probesets are presented. The signal intensity of the parental Balb/c-3T3 cells was set to 100%. **B.** Real-time PCR analysis. RNA samples used for microarray analysis were measured for Necdin mRNA expression using Real-time PCR with gene-specific primers and fluorescent-labeled probe (Taqman® Gene Expression Assays, Applied Biosystems). RNA expression was normalized to 18S rRNA. n = 3 independent experiments. **C.** Western. NIH3T3 cells stably expressing v-Src were seeded (2.5×10^5^) in 6 cm tissue culture plates in complete medium 24 h before transfection. Cells were then transfected with either 125 nM control siRNA or 100 nM or 125 nM STAT3 siRNA. At 48 h after transfection, total protein was harvested and equal amounts of total protein (100 ug) were loaded on a 10% SDS-polyacrylamide gel, electrophoresed and immunoblotted for Necdin (polyclonal, Abcam ab18554), phosphorylated STAT3 (p-STAT3, Cell Signaling 9131), total STAT3 (Santa Cruz, sc-482) and anti-actin (monoclonal, Sigma A-4551) proteins.

We then set out to verify the computational analysis and confirm whether Necdin is in fact a STAT3 target gene. NDN, the gene encoding Necdin, a negative growth regulator [31] and member of the MAGE family of melanoma-associated tumor antigens, was identified as one candidate STAT3-regulated gene. Five Affymetrix probesets corresponding to NDN are ranked in the list of the top 12 most significantly repressed probesets (Table 2). When compared with normal control cells, analysis of the microarray data demonstrated that NDN expression was consistently repressed in the cell lines expressing v-Src or STAT3-C, indicating that NDN is a candidate STAT3-regulated gene in both of these cell lines (Fig. 1A). Figure 1B. confirms that NDN mRNA expression is dramatically down-regulated in v-Src and STAT3-C expressing cells as measured by quantitative Real-Time PCR.

### Repression of Necdin mRNA Expression is STAT3 Dependent

NIH-3T3 cells stably expressing v-Src express high levels of active STAT3. These v-Src 3T3 cells were treated with either control siRNA or two different doses of STAT3-specific siRNA. Cells treated with control siRNA maintain high levels of STAT3 and have low levels of Necdin expression (Fig. 1C, lane 1). As expected, STAT3 siRNA effectively inhibited expression of total STAT3 (Fig. 1C, lanes 2 and 3). In these cells the expression of the STAT3 protein was inhibited in a dose-dependent manner, and Necdin expression was restored in a manner consistent with STAT3 knockdown in this cell line. These results suggest that repression of Necdin is dependent on activated STAT3.

### Activated STAT3 Binds To the Necdin Promoter *in vitro*

**Figure 2:**
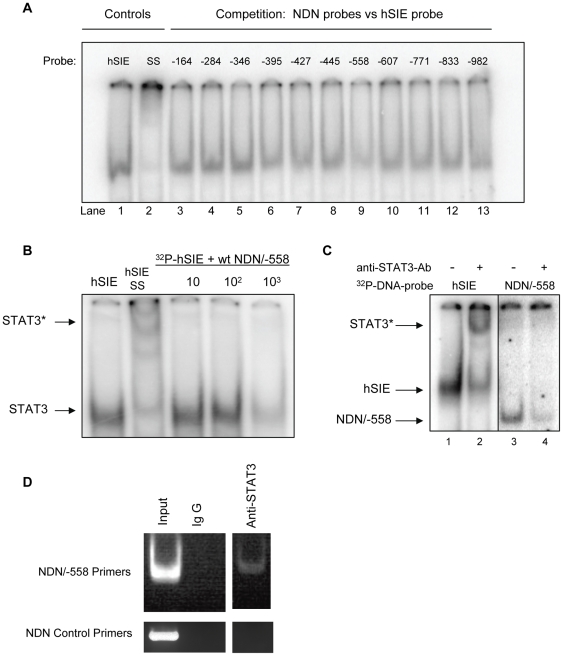
STAT3 binds directly to the Necdin promoter. **A.** Competition EMSA. Oligonucleotides for the sense and antisense strands of all putative STAT3-binding sites in the Necdin promoter were annealed and used to compete for STAT3 binding with ^32^P-labeled hSIE oligonucleotide using 5 ug of nuclear extract from Balb/c-3T3 cells stably transfected with v-Src as a source of activated STAT3. A candidate STAT3 DNA binding site in the mouse Necdin promoter was identified (position −558, relative to the translation initiation site). The high affinity STAT3 binding oligonucleotide, hSIE was used as positive control. *, “supershifting” was achieved using anti-STAT3 antibodies added to the reaction to confirm the presence of STAT3 in the complex. **B.** Competition EMSA. 3T3 v-Src nuclear extract was incubated with either ^32^P-labeled double stranded hSIE oligonucleotide (lanes 1 and 2) or with unlabeled, wild-type NDN/−558 oligonucleotide in a 10- to 10^3^-fold molar excess (lanes 3–5) prior to adding ^32^P-labeled hSIE oligonucleotide, to compete with hSIE for STAT3 binding. SS, supershift with anti-STAT3 antibodies. **C.** EMSA. 3T3 v-Src nuclear extract was incubated with the following ^32^P-labeled double-stranded oligonucleotides: hSIE (lanes 1 and 2), NDN/−558 (lanes 3 and 4), with and without anti-STAT3 antibodies. **D.** Chromatin immunoprecipitation assay (ChIP). Balb/3T3 v-Src cells expressing constitutively active STAT3 were used for ChIP. Briefly, after crosslinking histones to DNA by formaldehyde for 10 min, cells were collected and sonicated to shear DNA to an average length of 200–1000 bp. A portion of this material was used as a positive control for PCR (Input). The remaining sample was incubated with either anti-IgG or anti-STAT3 antibodies overnight and then immunoprecipitated using protein A-agarose. The histone-DNA complex was reverse cross-linked after several washing steps, and samples were subjected to PCR using specific primers surrounding the candidate STAT3-binding site at position −558 in the NDN promoter.

To determine whether STAT3 directly regulates Necdin transcription, we analyzed the sequence of the mouse NDN promoter [32] for potential STAT3 binding sites. STAT3 consensus sites have been defined as palindromic sequences with the common sequence 5′-TT(N_4–6_)AA-3′[49]. Our analysis identified several candidate STAT3 binding sites throughout the 1500 base pairs upstream of the transcriptional start site. Double-stranded oligonucleotide probes were generated for all the potential binding sites and tested in a competition EMSA (Fig. 2A) for their ability to compete for the binding of STAT3 against a high affinity variant of the STAT3 binding site in the c-*fos* promoter (hSIE) [50]. The oligonucleotide containing the putative binding site at position −558 relative to the transcription initiation site was identified as being able to compete most effectively with the hSIE probe (Fig. 2A, lane 9). Furthermore, we tested the ability of increasing amounts of non-radioactive NDN/−558 oligonucleotide to compete with the radiolabelled hSIE probe for binding of activated STAT3. As shown in Figure 2B, a high molar excess of unlabeled NDN/−558 is able to compete with ^32^P-hSIE for STAT3 binding.

A double stranded ^32^P-radiolabelled DNA oligonucleotide corresponding to the NDN/–558 sequence identified in the NDN promoter was then used in an EMSA to detect STAT3 DNA binding. The NDN probe, as well as the positive control probe, hSIE, were incubated with 5 ug nuclear extract from v-Src 3T3 cells and subjected to native gel electrophoresis. As shown in Figure 2C, activated STAT3 binds to the high affinity sequence in the hSIE oligonucleotide (lane 1), as well as to the sequence derived from the NDN promoter (lane 3). To confirm that STAT3 is contained in the protein complex binding to the oligonucleotides, the nuclear extract was pre-incubated with anti-STAT3 antibodies before adding the radiolabelled probe (“supershifted” bands, lanes 2 and 4). NDN/−558 shows a weaker STAT3 DNA binding activity compared to the artificial high-affinity STAT3 binding element hSIE and the addition of anti-STAT3 antibody partially blocks binding of the radioactive probe. This could result if the antibody recognition site and DNA binding domain in STAT3 were in close proximity, causing the antibody to partially obstruct binding of STAT3 to the probe.

### Binding of STAT3 to the NDN Promoter *in vivo*

To determine whether STAT3 could bind the Necdin promoter in intact cells, chromatin immunoprecipitation assays (ChIP) were performed in 3T3 v-Src cells using an antibody specific to STAT3. As shown in Figure 2D, PCR yielded Necdin promoter DNA immunoprecipitated with an anti-STAT3 antibody in the region of the −558 putative STAT3-binding site, but not at a control location on the NDN promoter. The specificity of this binding interaction is demonstrated by the lack of signal generated when a control antibody is used (anti-rabbit IgG). These data provide evidence that STAT3 can directly bind the Necdin promoter in intact 3T3 v-Src cells.

Since both the EMSA and ChIP assays suggest that STAT3 has the ability to bind to the NDN promoter both *in vitro* and *in vivo*, this provides further evidence that control of NDN expression by STAT3 occurs through a direct binding event at the promoter and that gene regulation primarily occurs at the level of transcription.

### Necdin Expression Is Repressed in Human Melanoma Cells

We next examined whether down-regulation of Necdin occurred in human tumor cells with activated STAT3. Expression of Necdin has been previously shown to be repressed in melanoma cells [51] so we examined whether this had a correlation with STAT3 activity.

**Figure 3:**
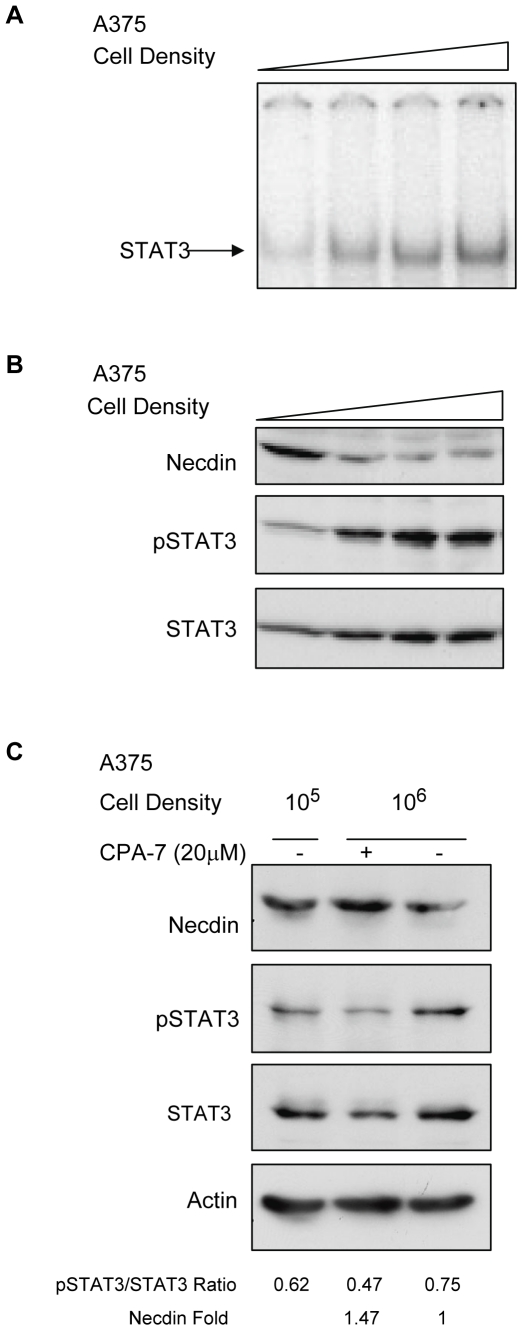
STAT3 downregulates Necdin expression in A375 human melanoma cells. A375 cells were plated at different densities (Fig. 3A and 3B: 1, 2.5, 5 or 7.5×10^5^ cells; Fig. 3C: 10^5^ and 10^6^ cells) in 10 cm plates and grown for 72 h. Nuclear extracts and total protein were collected. **A.** EMSA. Nuclear extracts from A375 cells were incubated with STAT3-specific hSIE ^32^P-labeled double stranded oligonucleotide. **B.** Western blot. Total protein extracts were harvested from A375 cells plated at different densities and equal amounts of total protein (100 ug) were loaded on a 10% SDS-polyacrylamide gel, electrophoresed and immunoblotted for Necdin, phosphorylated STAT3 (p-STAT3) and total STAT3 proteins. **C.** Western blot. A375 cells were plated at two different densities (10^5^ and 10^6^ cells) and allowed to adhere overnight. Plates seeded at 10^6^ cells were then treated with either DMSO or CPA-7 (20 umol/L) and all cells were grown for a further 48 h. Total protein was harvested and analyzed by Western blot. For densitometry, images were digitally scanned and optical density of the bands was quantified using Scion Image (Scion Corporation, Frederick, MD) and normalized to control. The ratio of pSTAT3 to total STAT3 was calculated for individual lanes.

STAT3 phosphorylation, STAT3 DNA-binding activity and total STAT3 levels have been shown to increase in A375 melanoma cells in a density-dependent manner in the absence of ligand [52]. A375 cells were plated at increasing density and allowed to grow for 72 h. Nuclear extracts were prepared and analyzed by EMSA. Figure 3A shows that DNA-binding of STAT3 increases with cell density as expected. We then analyzed total protein by Western blot for Necdin expression. Figure 3B shows that expression of total STAT3 and STAT3 phosphorylation is up-regulated in a density-dependent manner. Conversely, as STAT3 activation increases, Necdin expression is down-regulated at the protein level.

To confirm that the repression of Necdin expression is STAT3-dependent, A375 cells were plated at high density, and allowed to adhere overnight before being treated with either DMSO or the STAT3-inhibitor CPA-7 (20 umol/L) for 24 h [53]. Western blot analysis shows that when A375 cells are plated at low density (10^5^ cells), Necdin expression is high, whereas activated STAT3 levels are low (Fig. 3C, lane 1). Cells plated at high density (10^6^ cells), (Fig. 3C, lane 3) show higher levels of p-STAT3 and decreased expression of Necdin. Treatment of high density A375 cells with CPA-7 for 24 h inhibited STAT3 activation (Fig. 3C, lane 2), and Necdin levels in these cells are restored to high levels, comparable to cells plated at low density.

### IL-6 Represses Necdin Expression in Human Prostate Cancer Cells

**Figure 4:**
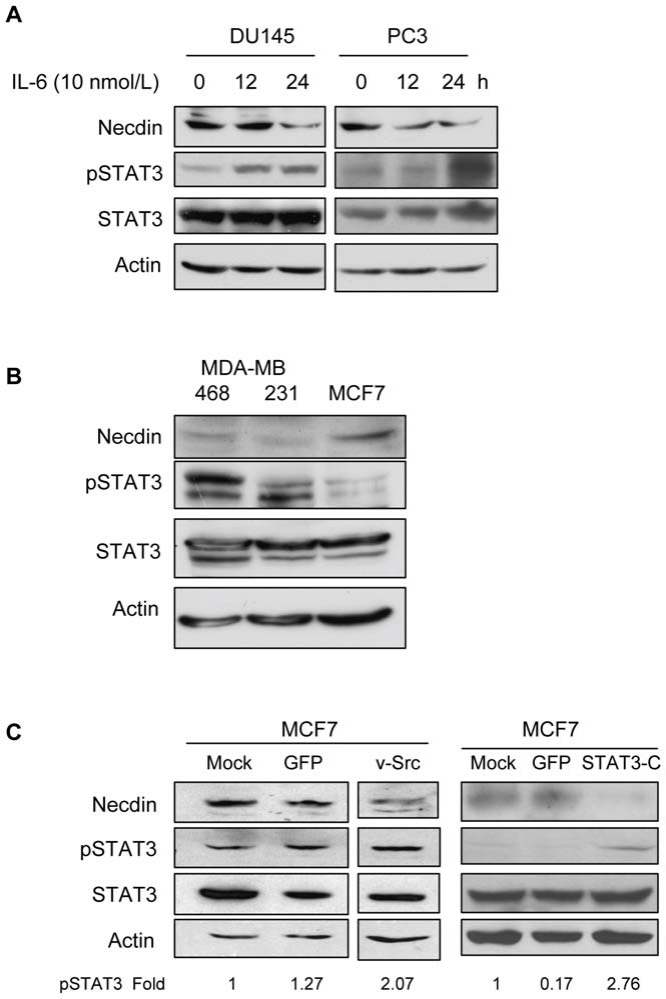
STAT3 activity down-regulates Necdin expression in human prostate cancer and breast cancer cell lines. **A.** Western blot. Total protein from DU145 and PC3 cells treated with IL-6 (10 nmol/L) for 12 or 24 hours was analyzed by Western blot. **B.** Total protein was harvested from MDA-MB-468, MDA-MB-231 and MCF-7 cells and subjected to Western blot analysis. **C.** MCF-7 cells were seeded and allowed to adhere overnight before being transiently transfected with control (GFP), pMvSrc or pRc/CMV-STAT3-C plasmids using Lipofectamine PLUS. Total protein was collected at 48 h post-transfection and subjected to Western blot analysis. For densitometry, images were digitally scanned and optical density of the bands was quantified using Scion Image (Scion Corporation, Frederick, MD) and normalized to control.

IL-6 acts as an autocrine growth factor in prostate cancer [54] and has been linked to progression of tumors [55]. IL-6 signals are transmitted via the JAK-STAT pathway from receptors on the cell surface to the target genes in the nucleus, involving phosphorylation and activation of STAT3 [56]. We therefore examined whether activation of STAT3 via IL-6 stimulation led to repression of Necdin expression in the prostate cancer cell lines DU145 and PC3. These cell lines harbor low levels of constitutively active STAT3 [57], [58], which can be further induced by stimulation with IL-6. Cells were serum starved for 3 h prior to treatment with IL-6 (10 nmol/L) for 12 or 24 h. Total protein was prepared and analyzed by Western blot. Figure 4A shows that IL-6 stimulation resulted in increased STAT3 activity within the cells and demonstrated corresponding down-regulation of Necdin expression upon IL-6 stimulation, in both cell lines. This confirms that IL-6 is capable of repressing Necdin expression via STAT3 in prostate cancer cells.

### Necdin Expression Correlates with STAT3 Activity in Human Breast Cancer Cells

Since EGFR and Src signaling pathways contribute to STAT3 activation in breast cancers [15], [34], we evaluated Necdin expression levels in human breast cancer cell lines with different levels of endogenous STAT3 activity. Figure 4B shows that p-STAT3 protein levels were high in MDA-MB-468 cells, slightly lower in MDA-MB-231 and very low in MCF-7 cells. Necdin protein expression inversely correlated with p-STAT3 levels, being expressed at a low level in MDA-MB-468 and MDA-MB-231 cells, but exhibited much higher expression in MCF-7 cells.

To test the hypothesis that constitutively activated STAT3 has a causal role in suppressing Necdin expression in tumor cells, we examined whether transient activation of STAT3 signaling could down-regulate Necdin expression. MCF7 cells express high levels of Necdin (Fig. 4C, lane 1 & 4), however when transiently transfected with v-Src or STAT3-C for 48 h, Necdin protein expression is inhibited. This demonstrates that even a transient 2-fold increase in STAT3 activation in these cells is sufficient to effectively repress the expression of Necdin (Fig. 4C, lane 3 & 6).

## Discussion

The transcriptional profile of a cell expressing constitutively-active STAT3 is predicted to be very different compared to a cell where STAT3 is under tight regulation. Our initial hypothesis was that STAT3 promotes widespread changes in global gene expression patterns, including both direct and indirect targets. We took a broad approach by studying global gene expression changes using microarray analysis in cells expressing constitutively-activated STAT3. With this approach we were able to confirm differential expression of several previously identified STAT3 target genes, as well as a novel target gene, with a wide range of biological functions and roles in multiple cellular pathways. These results suggest that STAT3 has a wider impact on cellular processes than demonstrated to date and that STAT3 also acts as a central coordinator of its own cellular signaling pathways.

Constitutive activation of STAT3 provides cancer cells with growth and survival advantages by activating multiple pathways within the cell, involving a broad range of genes. It has also been shown to repress the transcription of a handful of genes, including p53 [44] and nitric oxide synthase [45]. Few other genes have been identified to date that are negatively regulated by STAT3. In this study, we identified Necdin as a novel STAT3 target gene whose expression is repressed when STAT3 is constitutively activated. Our studies indicate that constitutively active STAT3 directly causes down-regulation of Necdin at the transcriptional level. We also demonstrated that Necdin expression is repressed in several tumor cell types, including melanoma, prostate and breast cancer cell lines, and is inversely correlated with STAT3 activity. This suggests that Necdin is a physiological target gene of STAT3.

A recent study published by Chapman and Knowles [33] stated that down-regulation of Necdin occurs in both carcinoma cell lines and primary tumors, suggesting that Necdin may have a tumor suppressor role. Our results confirm the data reviewed in this paper and indicate that Necdin is a candidate for further study in this role and could represent a novel cancer therapeutic target. Repression of Necdin expression by STAT3 may play an important role in regulating the cell cycle and proliferation in human cancer cells, which has the potential to give tumor cells a growth advantage. Necdin is a negative growth regulator, capable of interacting with E2F1, resulting in inhibition of E2F1 target gene expression and consequent growth inhibitory effects [59]. Two reports have previously demonstrated that Necdin expression is down-regulated in melanoma [51] and a drug-resistant ovarian cancer cell line [60]. Thus far, no role for Necdin in oncogenesis has been confirmed; however, our results suggest that repression of Necdin expression by STAT3 may be one mechanism which could potentially contribute to a growth advantage of tumor cells and is of interest for further analysis.

In summary, STAT3 has been shown to up-regulate expression of multiple genes involved in cell growth and metabolism, as well as protein transport, localization, cell adhesion and cytoskeletal remodeling. This study also suggests that STAT3 may exert its oncogenic effect not only by up-regulating transcription of genes involved in promoting growth and proliferation, but also by down-regulating expression of negative regulators of the same cellular processes, such as Necdin. The use of pharmacological agents to inhibit STAT3 activity may lead to a rebalancing of the signaling pathways regulating cell growth and lead to inhibition of tumor progression [5], [61]–[64].

## Materials and Methods

### Cell Culture and Reagents

Balb/c-3T3 mouse fibroblasts, v-Src 3T3 cells [50] and STAT3-C 3T3 cells were grown in DMEM/10% bovine calf serum supplemented with 1% penicillin and streptomycin. MDA-MB-231, MDA-MB-468 and MCF7 cells were obtained from the American Type Culture Collection (ATCC) and maintained in DMEM/10% fetal bovine serum supplemented with 1% penicillin and streptomycin. DU145, PC3 and A375 cells were obtained from ATCC and maintained in RPMI/10% fetal bovine serum supplemented with 1% penicillin and streptomycin. CPA-7 was a generous gift from Dr. Nick Lawrence. IL-6 was obtained from BD Pharmingen (San Jose, CA, USA).

### Expression Vectors

pRc/CMV-STAT3C-Flag (constitutively activated STAT3) was a generous gift from J. Bromberg and J. Darnell [36]. The v-Src plasmid expression vector, pMvSrc, has been described previously [65]. Transient transfections were performed using Lipofectamine PLUS (Invitrogen, Carlsbad, CA, USA) according to the manufacturer's protocol.

### siRNA Transfections

siRNA directed specifically against STAT3 and a non-targeting control siRNA were obtained from Dharmacon RNA Technologies (Chicago, IL, USA). Cells were transfected using RiboJuice transfection reagent (Novagen, Gibbstown, NJ, USA) as per the supplier's instructions. At 48 h after initial transfection, non-adherent cells were washed off and the remaining cells were harvested for Western blot.

### RNA Extraction and Microarray Analysis

At each of 3 passages, cells from the five dishes were pooled and total RNA was extracted using Trizol (Invitrogen) and further purified using the RNeasy kit (Qiagen, Valencia, CA, USA) according to the supplier's instructions. Total RNA (1 ug) was then used for microarray analysis using Affymetrix Mouse Genome 2.0 GeneChips, according to the manufacturer's protocol.

### Microarray Data Analysis

The CEL files were normalized using Robust Multichip Average (RMA) [66]. In order to identify changes in gene expression caused by v-Src or STAT-3C expression, CEL files for three Balb/c-3T3 control chips were either normalized with the CEL files for the three v-Src chips or normalized separately with the CEL files for the three STAT3-C chips. The expression sets were then exported to a Microsoft Excel spreadsheet, formatted for analysis by the Significance Analysis of Microarrays (SAM) add-in tool for Excel [37]. SAM was performed twice: first to identify differentially expressed genes between control Balb/c-3T3 cells and Balb/c-3T3 cells expressing pMvSrc, then again to identify differentially expressed genes between control Balb/c-3T3 cells and Balb/c-3T3 cells expressing pRcCMV-STAT3-C.

The options selected for SAM analysis were as follows: Response Type: two-class, unpaired data (Class 1 – Balb/c-3T3, Class 2 – v-Src or STAT3-C); Data logged: logged (base 2); Weblink Option: Accession number; Number of Permutations: 100; Imputation engine: N/A – no missing data in experiment; Random number seed: generate random number seed. This produced a list of Affymetrix probeset IDs differentially expressed in cells expressing v-Src and also for STAT3-C as compared to control cells. We accepted all probesets identified by SAM as differentially regulated by at least 1.5-fold.

Probeset IDs in common between the two lists were identified using the Excel VLOOKUP function. The probesets identified were then processed by Affymetrix NetAffx to yield a list of genes. SAM analysis generates a Score (t-statistic) for each probeset on each list. Probesets common to both lists were ranked using the average of the two Score values generated from the v-Src and STAT3-C SAM analysis.

The microarray data have been deposited in the Gene Expression Omnibus (GEO) Database at http://www.ncbi.nlm.nih.gov/geo (GEO accession no. GSE22251).

### Computational Analysis of Microarray Data

We analyzed and categorized the differentially expressed genes identified by SAM using the Functional Annotation tool in the DAVID Bioinformatics Database [44], [45]. Pathway Analysis was carried out using the MetaCore Analysis Suite v 5.2 build 17389 GeneGO Maps program (GeneGO, Inc, New Buffalo, MI) to identify signaling pathways that were enriched in the list of differentially expressed genes.

### Quantitative Real-time PCR

For real-time PCR, total RNA was isolated as detailed above. An aliquot of the same RNA used for microarray analysis, was used for Real-Time PCR using TaqMan® Gene Expression Assays (Applied Biosystems, Foster City, CA, USA) according to the manufacturer's instructions. TaqMan® Gene Expression Assay Mm02524479_s1* was used to analyze Necdin expression and Eukaryotic 18S rRNA (4319413E, Applied Biosystems) was used as an endogenous control. Data were analyzed using SDS software version 2.2.2 and exported into an Excel spreadsheet.

### Western Blot Analysis

Whole cell lysates were prepared in boiling sodium dodecyl sulphate (SDS) sample buffer and equal amounts (100 ug) of total protein were run on a 10% SDS-polyacrylamide gel. The proteins were transferred to nitrocellulose membrane, washed with PBS/0.2% Tween 20, and incubated in 1× PBS/0.2% Tween 20/5% milk overnight with anti-phospho-STAT3 antibody (Cat. #9131, Cell Signaling, Boston, MA, USA), or anti-STAT3 antibody (sc-482, Santa Cruz Biotechnology, Santa Cruz, CA) or anti-Necdin antibody (ab18554, Abcam, Cambridge, MA, USA). The membrane was then washed with PBS/0.2% Tween 20, incubated for 1 h at room temperature with alkaline phosphatase–linked anti-rabbit secondary antibodies, and visualized using ECL Western Blotting Detection Reagents (Amersham, Pittsburgh, PA, USA). For detection of ß-actin, the blot was stripped with stripping buffer [2% SDS, 62.5 mmol/L Tris (pH 6.8), 0.7% ß-mercaptoethanol] and re-blotted with anti-ß-Actin (A5441, Sigma) for 1 h at room temperature and visualized as described. Bands were detected by autoradiography. For densitometry, images were digitally scanned and optical density of the bands was quantified using Scion Image (Scion Corporation, Frederick, MD) and normalized to control.

### Chromatin Immunoprecipitation

Chromatin immunoprecipitation was performed using a kit from Upstate as described by the supplier. Briefly, 2 million v-Src 3T3 cells were treated with formaldehyde for 10 minutes at room temperature. Cells were collected by scraping, lysed and the DNA sheared by ultrasonication (Bioruptor XL, Diagenode). Immunoprecipitations were performed with the following antibodies (4.0 ug): anti-total STAT3 (sc-482, Santa Cruz Biotechnology), and anti-rabbit Ig G (sc-2027, Santa Cruz Biotechnology) as a control. Subsequently, cross-links were reversed, and bound DNA was purified. PCR was performed using Necdin specific primers: Fwd: 5′-CATgAgAgACTgTTAggTATC-3′ and Rev: 5′-CTATAgATTTgggCTCTCCAT-3′.

### Nuclear Extract Preparation and EMSA

For the detection of DNA-binding activity of STAT3 by EMSA, nuclear protein extracts were prepared using high-salt extraction as described previously [34]. Nuclear protein (5 ug) from Balb/c-3T3 v-Src cells was incubated with ^32^P-radiolabelled dsDNA oligonucleotides using a high-affinity variant of the sis-inducible element (hSIE; sense strand, 5′-AgCTTCATTTCCCTgAAATCCCTA-3′) derived from the c-fos gene promoter, which binds activated STAT3 and STAT1 proteins as a positive control [52]. In addition, wild-type oligonucleotide probes derived from the Necdin gene promoter were used, including the following (STAT3 consensus DNA-binding sequence italicized): wild-type Necdin/–558 (sense strand, 16-mer), 5′-CTAC*TTCTAgAA*-3′. Supershift assays were performed using anti-STAT3 polyclonal antibodies (C20X, Santa Cruz Biotechnology) to identify STAT3. 2 uL of the concentrated STAT3 antibody was pre-incubated with 5 ug nuclear protein for 20 min at room temperature before adding the radiolabelled probe (30 min, 30°C). Samples were then separated by non-denaturing PAGE and detected by autoradiography.

## Supporting Information

Table S1 **Top 50 Most Significant Probesets Over-Expressed Common to vSrc and STAT3C.** The top 50 genes ranked by Average Score identified by SAM as being upregulated in common by vSrc and STAT3-C.
(XLS)

Table S2 **Top 50 Most Significant Probesets Under-Expressed Common to v-Src and STAT3-C.** The top 50 genes ranked by Average Score identified by SAM as being downregulated in common by vSrc and STAT3-C.
(XLS)

Table S3 **Functional Enrichment (based on GO Biological Process) in Genes Differentially Expressed in Common by v-Src and STAT3-C using DAVID.** The top 10 functionally enriched categories ranked by p-Value for GOTERM_BP_3 identified by DAVID. ^i^ Column expands to show all differentially expressed Affymetrix probesets included in this category.
(XLS)
